# Supplementary material for: Biomarker-Based Analysis of Pain in Patients with Tick-Borne Infections before and after Antibiotic Treatment
Source: Antibiotics (Basel). 2024 Jul 25;13(8):693. doi: 10.3390/antibiotics13080693 (PMC11350843; doi:10.3390/antibiotics13080693)
Supplement: Supplementary file 1 [file antibiotics-13-00693-s001.zip › antibiotics-3098525-supplementary.pdf]

Biomarker-Based Analysis of Pain in Patients with Tick-Borne Infections before and after Antibiotic Treatment

Kunal Garg 1,†, Abbie Thoma 2,†, Gordana Avramovic 2, Leona Gilbert 1, Marc Shawky 3, Minha Rajput Ray 4 and John Shearer Lambert 2,5,6,*

1 Te?ted Oy, 40100 Jyväskylä, Finland; kunal.garg@tezted.com (K.G.); leona.gilbert@tezted.com (L.G.)
2 Department of Infectious Diseases, Catherine Mc Auley Education & Research Centre, Mater Misericor-diae University Hospital, 21 Nelson Street, Dublin 7, D07 A8NN Dublin, Ireland; abbie.thoma@ucdconnect.ie (A.T.); gavramovic@mater.ie (G.A.)
3 Université de Technologie de Compiègne, Costech Laboratory, Alliance Sorbonne Université, Centre de Recherches, 60203 Compiègne, France
4 Curaidh Clinic: Innovative Solutions for Pain, Chronic Disease and Work Health, Perth PH2 8EH, UK; drminha@curaidh.com
5 Catherine Mc Auley Education & Research Centre, University College Dublin, 21 Nelson Street, Dublin 7,
D07 A8NN Dublin, Ireland
6 Infectious Diseases Department, The Rotunda Hospital, D01 P5W9 Dublin, Ireland
* Correspondence: jlambert@mater.ie
† These authors contributed equally to this work.

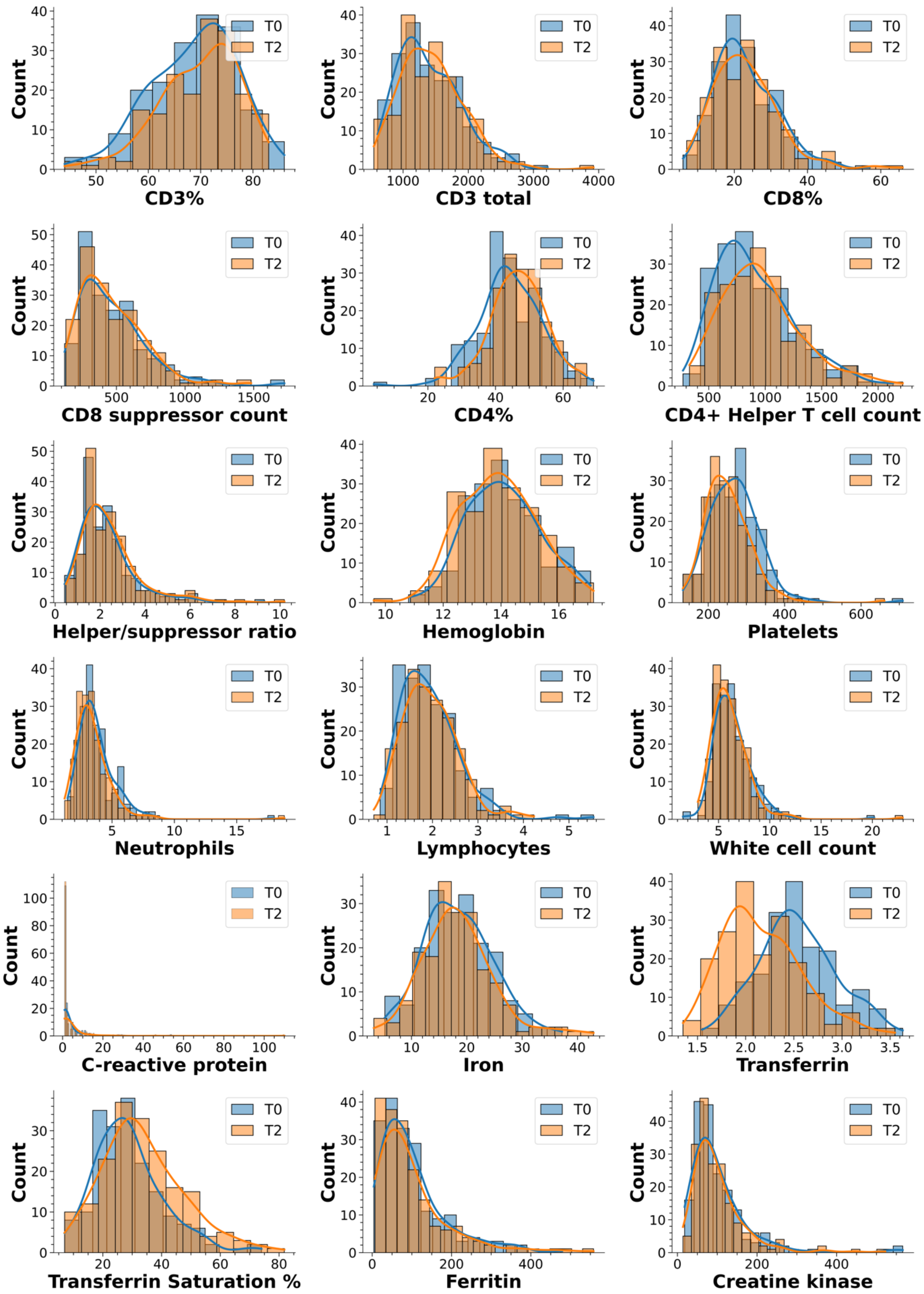

Figure S1. Overall distribution of all biomarkers at time points T0 and T2.

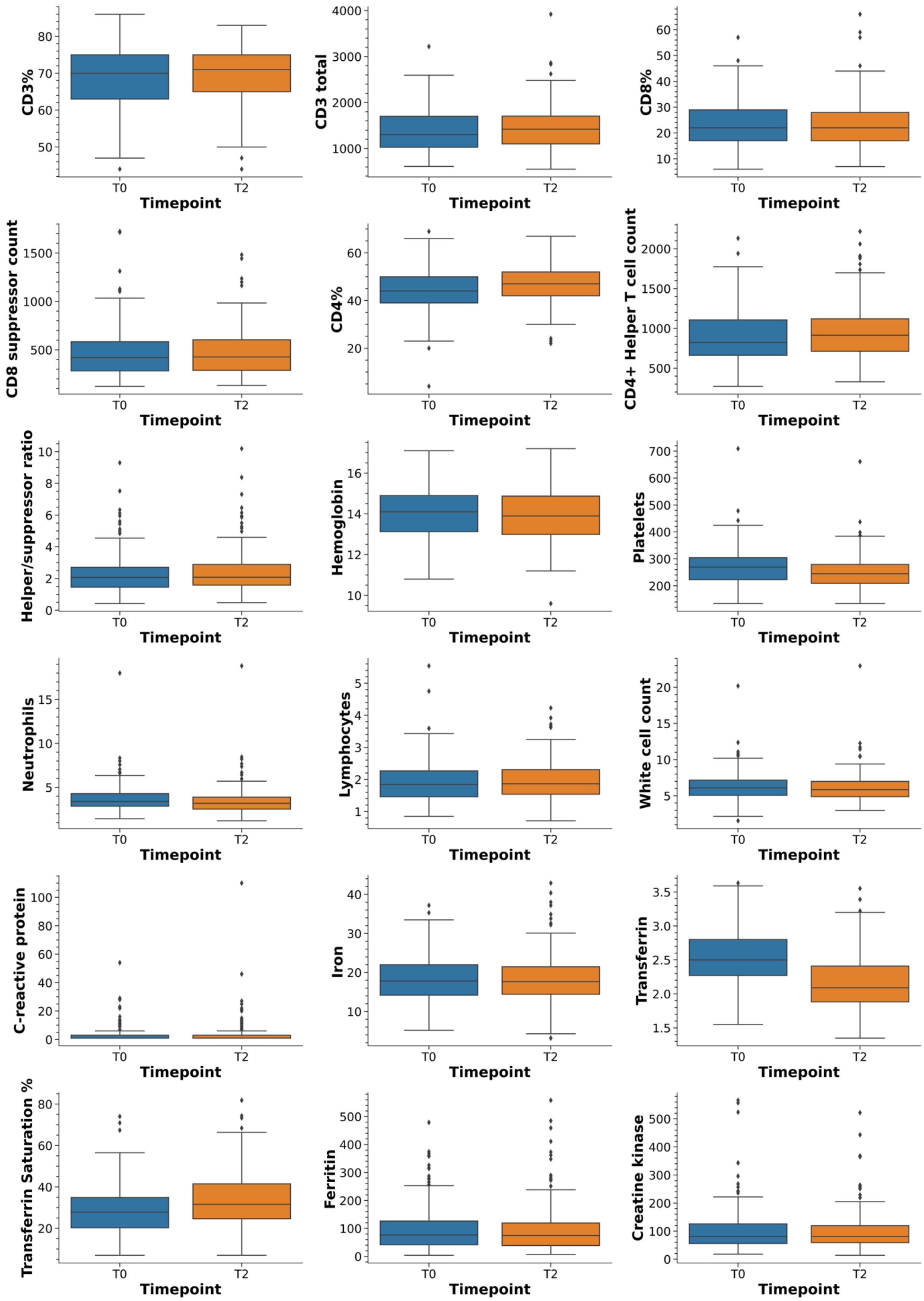

Figure S2. Change in median values for all biomarkers at time points T0 and T2.

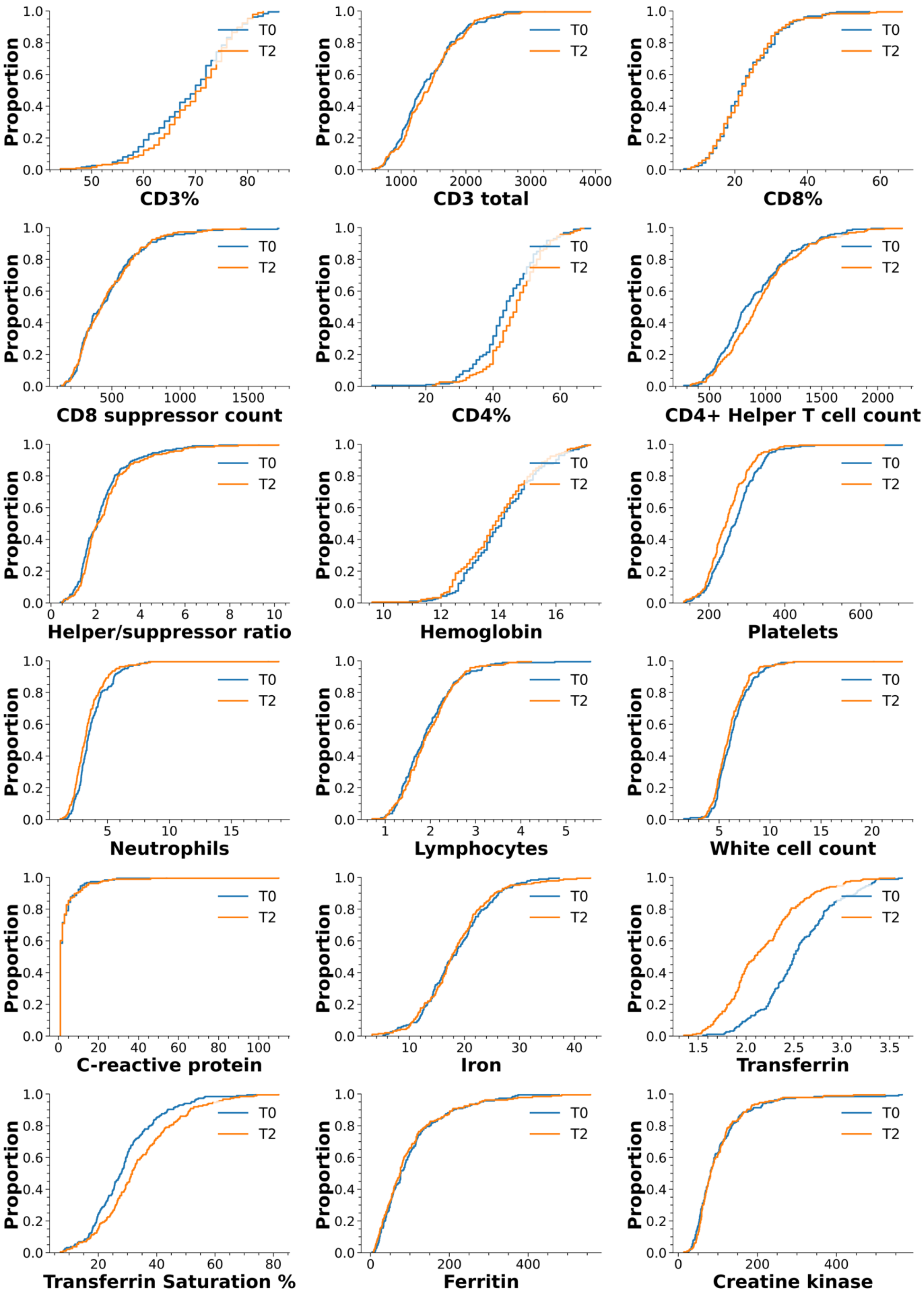

Figure S3. Change in distribution for all biomarkers at time points T0 and T2.

Table S1: Differences in median values of biomarkers at T0 and T2 for pain ratings ranging from 1 to 10. The Mann-Whitney U test was used to evaluate the statistical significance of changes in median values across each pain rating. Pain levels with statistically significant differences (*p*-value ≤ 0.05) are highlighted in green.

**Pain Rating**	**1**	**2**	**3**	**4**	**5**	**6**	**7**	**8**	**9**	**10**
Transferrin median T0	2.45	2.53	2.31	2.46	2.48	2.59	2.49	2.51	2.61	2.39
Transferrin median T2	2.07	2.12	2.02	2.01	2.29	2.11	2.12	1.96	2.09	2.12
Transferrin *U* statistic	51.50	40.50	149.50	76.50	217.00	830.50	606.50	314.50	64.00	41.00
**Transferrin *p* value**	0.39	0.32	0.16	0.08	0.06	0.00	0.00	0.03	0.03	0.08
CD4% median T0	41.00	28.00	39.50	47.50	48.50	44.00	40.00	48.50	42.50	45.00
CD4% median T2	45.50	46.50	47.00	45.00	45.50	44.50	52.00	44.00	47.00	52.00
CD4% *U* statistic	36.00	20.00	34.00	60.00	172.00	530.50	129.50	284.50	26.50	11.50
**CD4% *p* value**	0.79	0.53	0.00	0.59	0.63	0.91	0.00	0.13	0.49	0.27
Platelets median T0	300.00	264.00	276.50	236.50	261.50	273.00	260.00	256.00	274.00	272.00
Platelets median T2	252.00	250.00	226.00	218.00	243.00	263.00	254.00	255.00	245.00	184.00
Platelets *U* statistic	57.00	31.00	178.00	61.00	176.50	592.00	426.00	222.00	42.50	45.00
**Platelets *p* value**	0.21	0.84	0.01	0.54	0.53	0.51	0.71	0.99	0.64	0.03
Neutrophils median T0	3.43	3.04	3.93	2.64	3.49	3.59	3.34	3.34	3.46	3.99
Neutrophils median T2	3.29	3.09	3.30	2.94	2.99	3.47	2.86	3.34	4.25	4.86
Neutrophils *U* statistic	45.00	23.00	136.00	43.00	200.50	601.50	541.50	199.50	30.00	15.50
**Neutrophils *p* value**	0.73	0.71	0.38	0.55	0.17	0.43	0.03	0.62	0.69	0.48
%Trans sat median T0	22.00	27.10	28.85	33.85	31.50	25.35	29.90	29.45	27.95	20.50
%Trans sat median T2	29.85	29.40	35.90	29.30	32.80	31.90	31.60	29.35	19.80	25.70
%Trans sat *U* statistic	31.50	19.50	81.00	61.50	145.00	441.50	316.00	215.50	55.00	18.00
**%Trans sat *p* value**	0.54	0.51	0.25	0.51	0.74	0.21	0.17	0.91	0.15	0.65

Pain rating

Pain rating	10 T0	10 T2	9 T0	9 T2	8 T0	8 T2	7 T0	7 T2	6 T0	6 T2	5 T0	5 T2	4 T0	4 T2	3 T0	3 T2	2 T0	2 T2	1 T0	1 T2
10	84	3																		
9	87	20	11	7																
8	89	20	12	14	15	18														
7	89	19	10	10	10	17	23	9												
6	87	18	12	9	12	18	23	9	12	8										
5	91	17	10	12	10	22	25	12	10	12	10	12								
4	94	13	14	9	14	22	30	11	16	12	15	12	14	9						
3	92	13	16	9	16	23	31	10	17	11	6	11	7	10	8	12				
2	89	18	8	12	8	18	24	11	9	11	10	11	9	15	8	12	7	12		
1	85	21	10	15	10	23	19	15	10	15	10	15	12	17	13	16	12	15	14	17

**Euclidian distance scale**	0	10	20	30	40	50	60	70	80	90	100

Figure S4. Heatmap demonstrating Euclidean distances between individual pain clusters (1-10) and their biomarker values for transferrin, CD4%, platelets, neutrophils, and transferrin saturation % using the K-Nearest Neighbours (KNN) technique. These Euclidean distances are normalized to absolute values and considered unitless. For example, in the case of row 3 (pain level = 3) and column 7 (pain level = 7), the value “31” at T0 represents the distance between the said biomarker profiles (of the patient groups reporting levels 3 and 7 of pain, respectively. The diagonal represents the distances between patient groups declaring the same pain level. As the diagonal values are mostly the smallest, we may conclude that patients reporting the same pain level have the closest biomarker profiles, with pain on a scale ranging from 1 to 10. The distances are symmetric to the diagonal. On the other hand, patients reporting the maximum pain level (10) at T0 have their biomarker profiles showing higher distances from other patients, making them a distinct group. However, at T2, we note that for pain level 10 clusters, the distances between biomarker profiles have substantially decreased. Moreover, overall inter-distances between biomarker profiles have notably decreased at T2, which may indicate a normalization progression.
